# ZnO/Cu_2_S/ZnO Multilayer Films: Structure Optimization and Its Detail Data for Applications on Photoelectric and Photocatalytic Properties

**DOI:** 10.3390/ma10010037

**Published:** 2017-01-05

**Authors:** Zhenxing Wang, Feng Xu, He Wang, Hai-Ning Cui, Haishui Wang

**Affiliations:** 1 Department of Optical Information Science and Technology, College of Physics and College of Zhaoqing, Jilin University, Zhaoqing 526061, China; yimingtongtian@163.com (Z.W.); xufeng_jlu@126.com (F.X.); whe15@mails.jlu.edu.cn (H.W.); 2 Department of Optical Information Science and Technology, College of Electronic Science and Engineering, Jilin University, Changchun 130021, China; 3 Department of Applied Chemistry, School of Chemistry and Chemical Engineering, South China University of Technology, Guangzhou 510641, China; wanghsh@scut.edu.cn

**Keywords:** multilayer, thin films, sputtering, electrochemical properties, optical properties

## Abstract

Monolayer Cu_2_S and ZnO, and three kinds of complex films, Cu_2_S/ZnO, ZnO/Cu_2_S, and ZnO/Cu_2_S/ZnO, were deposited on glass substrates by means of radio frequency (RF) magnetron sputtering device. The impact of the thickness of ZnO and Cu_2_S on the whole transmittance, conductivity, and photocatalysis was investigated. The optical and electrical properties of the multilayer were studied by optical spectrometry and four point probes. Numerical simulation of the optical transmittance of the multilayer films has been carried out in order to guide the experimental work. The comprehensive performances of the multilayers as transparent conductive coatings were compared using the figure of merit. Compared with monolithic Cu_2_S and ZnO films, both the optical transmission property and photocatalytic performance of complex films such as Cu_2_S/ZnO and ZnO/Cu_2_S/ZnO change significantly.

## 1. Introduction

ZnO films attract much attention of researchers as potential ultraviolet (UV), blue and other visible optical device materials owing to their wide and direct band gap, about 3.1–3.3 eV [1,2,3,4]. Additionally, polycrystalline ZnO has been found for numerous interesting applications, such as piezoelectric transducers and transparent conducting films [5,6,7,8,9]. However, the n-type ZnO is limited in its application because it is electrically too large in various environments. We need to develop a new material with better optical properties and lower resistivity than that of pure ZnO. At present, the main research objective of developing new materials for transparent conductive oxides films is to achieve higher transmittance and lower resistivity in the spectrum visible range. Recently, a combination of dielectric, semi-conductor and metal were used to produce highly transparent conducting oxides [10,11,12,13]. p-type semiconductors are important materials for developing optoelectronic devices such as large area flat-panel displays, solar cells and light-emitting diodes. To our knowledge, much attention is focused on the Cu_2_O/ZnO heterojunction for solar cells and photocatalysis studies [14,15], and there are few reports involving Cu_2_S/ZnO multilayers for the above studies. As a kind of p-type semiconductor, Cu*_x_*S attracts much attention of researchers because of electrical and optical properties. Cu*_x_*S has five crystalline phases, Cu_2_S, Cu_1.95_S, Cu_1.8_S, Cu_1.75_S and CuS. From this set of copper sulfides, Cu_2_S is considered a promising material as absorber of visible light, due to its band gap energy, 1.2–2.4 eV [16,17]. Besides, the combination of p-type and n-type materials leads to application of p-n junction devices in the future. Furthermore, ZnO and Cu_2_S complex films have a good photocatalytic performance [18]. They can completely destruct the undesirable contaminants in both liquid and gaseous phase using solar or artificial light illumination [18,19]. As we know, the efficiency of photocatalytic reaction is determined by the absorption ability of light and high separation rate of photo-induced electron-hole pair. There are many different Cu_2_S fabrication methods, such as electrodeposition, sol-gel, and hydrothermal, however the use of radio frequency (RF) magnetron sputtering is very rare [20]. In this paper, films of three different combinations (Cu_2_S/ZnO, ZnO/Cu_2_S, and ZnO/Cu_2_S/ZnO) were fabricated by RF magnetron sputtering. Their transparent conductive and photocatalytic properties were investigated, compared and discussed. In order to get a better sandwich structure, numerical simulation and fabrication of ZnO/Cu_2_S/ZnO multilayer were carried out for optimizing the multilayer system. The characteristic of the layer microstructure consisting of ZnO and Cu_2_S and the influence of the ZnO and Cu_2_S layer thicknesses are the focus of the study and discussion.

## 2. Experimental Section

Cu_2_S/ZnO, ZnO/Cu_2_S and ZnO/Cu_2_S/ZnO layered films were deposited on glass substrates using a Zn target (99.99% purity, 50 mm diameter) and a Cu_2_S target (99.9% purity, 50 mm diameter) by RF magnetron sputtering. The glass substrates were ultrasonically cleaned in acetone and alcohol, and rinsed in deionized water in sequence then dried in flowing nitrogen gas. High purity argon (Ar, 99.99% purity) and oxygen (O_2_, 99.99% purity) were used as the sputtering and reactive gases, respectively. The working chamber was pumped down to 5 × 10^−4^ Pa before deposition. The distance between the targets and substrates was 10 cm. The ZnO was deposited in O_2_ and Ar mixed atmosphere with O_2_/Ar pressure ratio 1:4, correspondent to a deposition pressure of 0.6 Pa. The Cu_2_S film was deposited in a pure Ar atmosphere, with a deposition pressure of 0.6 Pa. For both layers, the applied sputtering power was 100 W. For each film combination, the sets of films were produced under the following conditions:

(1) For Cu_2_S/ZnO combination, the ZnO layer was deposited with a thickness around 60 nm, and then the Cu_2_S was deposited on the ZnO layer. The thickness of Cu_2_S varied from 0 nm to 120 nm.
(2) For ZnO/Cu_2_S combination, the ZnO was deposited on the Cu_2_S layer whose thickness was 60 nm. The ZnO layer thickness varied from 0 nm to 60 nm.
(3) For the ZnO/Cu_2_S/ZnO combination, bottom ZnO thickness was fixed at 60 nm, and the Cu_2_S layer thickness was fixed at 60 nm. The top ZnO layer varied from 0 nm to 60 nm.

The thickness and growth rate of the films were monitored using the quartz crystal oscillator film-thickness apparatus (FTM-V, Taiyao Vacuum Tech, Shanghai, China). The UV-visible and near-infrared photometer was used to measure the transmittance in the visible and near-infrared regions with the automatic source selection mode. All the transmittance spectra are in the glass and air ground. The sheet resistance of films was measured using the four-point probemethod (RTS-8, Four Probes Tech, Guangzhou, China).

The photodegradation of methyl orange was chosen to evaluate the photocatalytic activity of our samples. The photocatalytic reaction was carried out in a beaker containing the prepared sample (2.5 × 2.5 cm^2^) and 30 mL of 10^−4^ M methyl orange aqueous solution. The solution was irradiated with a 175 W mercury lamp with central wavelength of 365 nm. The distance between the UV light source and the sample was set 20 cm. The temperature of methyl orange aqueous solution was kept at 30 °C during experiment process. Each 10 min of radiation, the concentration of methyl orange was examined using the AvaSpec-2048 type dual-channel fiber optical spectrometer by collecting the absorbance of the methyl orange. The photocatalytic reactivity of different kinds of films is quantified by the ratio of the residual concentration (C) to the initial concentration (Co) of methyl orange, denoted as 1-C/Co.

Surface morphology was observed using a field emission scanning electron microscope (FE-SEM, XL 30, Philips, Eindhoven, Holland). The phase characterization was carried out with X-ray diffractometer (XRD) using Cu Kαradiation (λ = 0.15418 nm) in the θ–2θ scan mode. X-ray tube voltage and current were set at 50 kV and 150 mA, respectively. The scanning range of the diffraction angle 2θ was varied from 10° to 80° at 0.02° step size, and the scanning speed was 4°/min.

## 3. Results and Discussion

### 3.1. Monolithic Film

#### 3.1.1. ZnO/Glass

**Figure 1:**
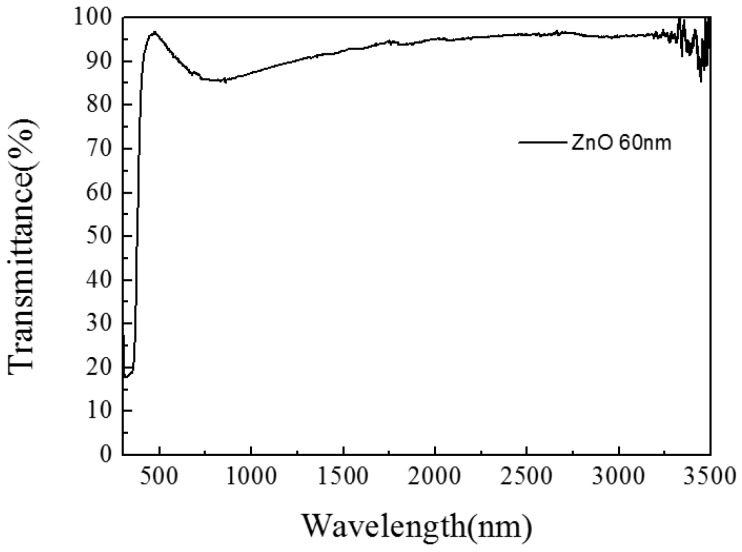
The transmittance diagram of ZnO/glass complex films for different thickness.

Before investigating the optical properties of the complex film, transmission, conductivity and catalytic tests on the ZnO/glass thin film have been carried. The results of the ZnO/glass thin film are as follow: in Figure 1, the transmission of ZnO/glass thin film after the wavelength is larger than 500 nm is very high, above 90%; the sheet resistance value of ZnO/glass thin film is very big (more than 1000 Ω/□) (Ω/□ is the unit of Sheet resistance. □ is equivalent to square) and this lead to a poor conductivity of film.

**Figure 2:**
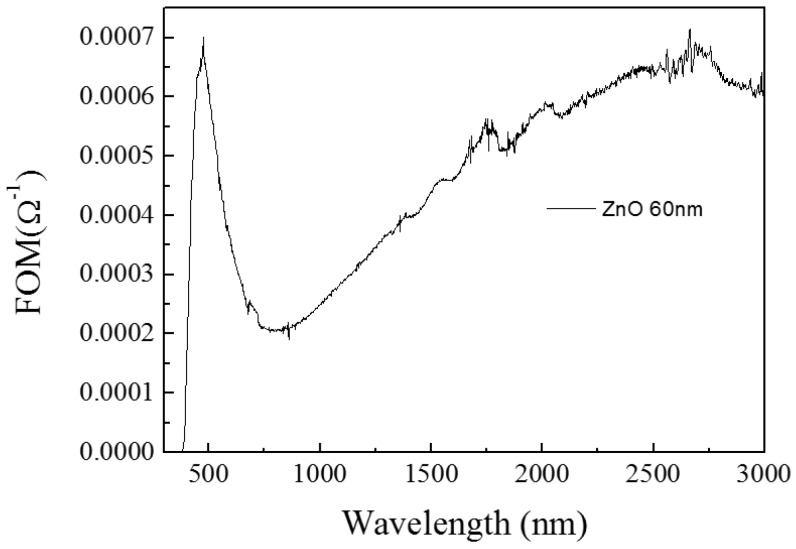
The figure of merit of ZnO/glass complex films for different thickness.

**Figure 3:**
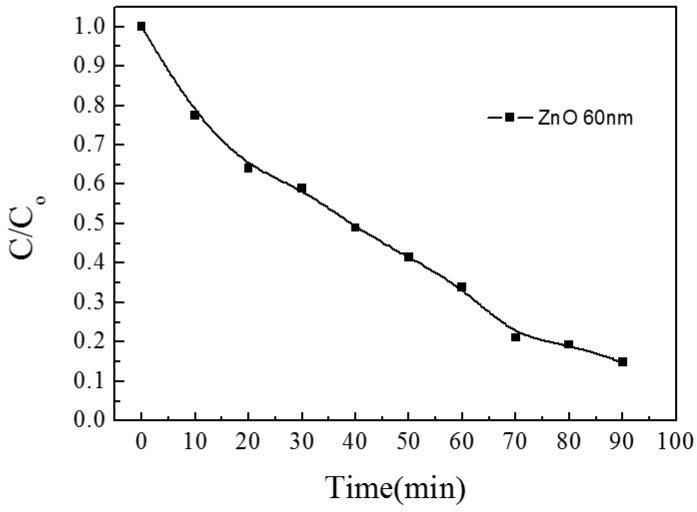
The photocatalytic activity of ZnO/glass complex films for different thickness.

To evaluate the TCO films and compare their photoelectric performance, the figure of merit (FOM) is introduced. FOM = *T*^10^/*R*s was defined by Haacke [21], where *T* is the optical transmittance and *R*s is the sheet resistance. In Figure 2, the law of the change of FOM value is similar to the change of transmission; in Figure 3, the photocatalytic effect of ZnO/glass thin film is very good, and its value can reach 80%.

**Figure 4:**
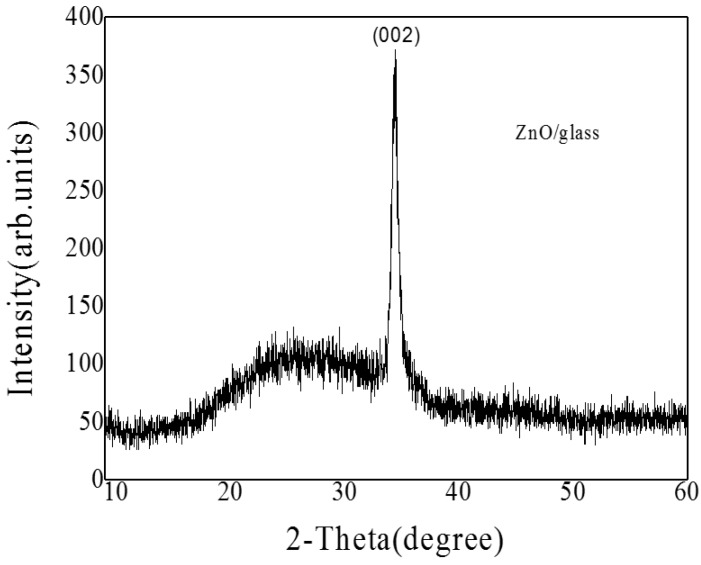
The XRD patterns of ZnO/glass films.

The crystalline structures of the ZnO/glass were estimated by XRD measurements, and the patterns are shown in Figure 4. The only detected diffraction peak locates in 2θ = 34.42°, corresponding to ZnO(002), the lattice constant of ZnO is 0.2602 nm.

#### 3.1.2. Cu_2_S/Glass

**Figure 5:**
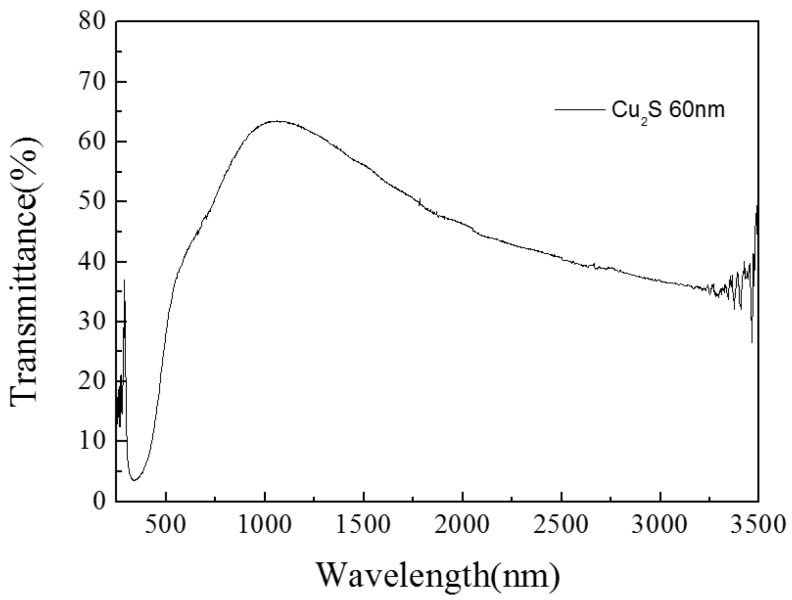
The transmittance diagram of Cu_2_S/glass complex films for different thickness.

**Figure 6:**
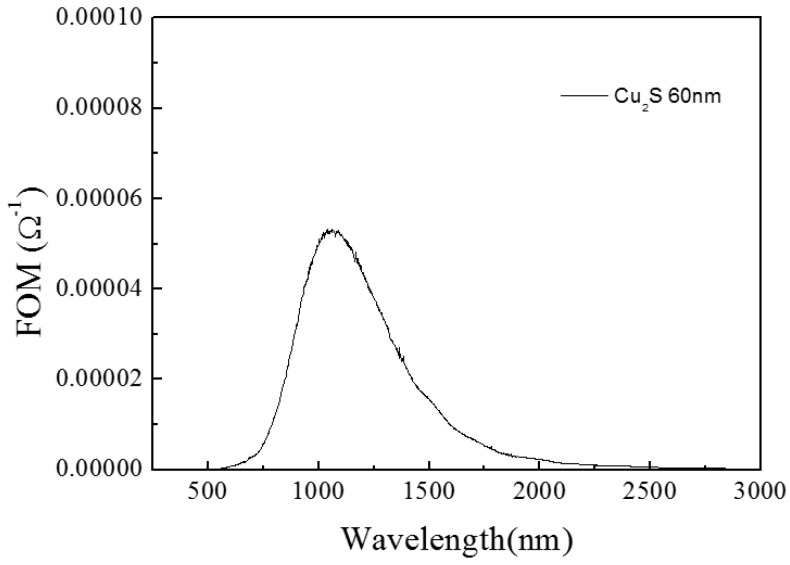
The figure of merit of Cu_2_S/glass complex films for different thickness.

**Figure 7:**
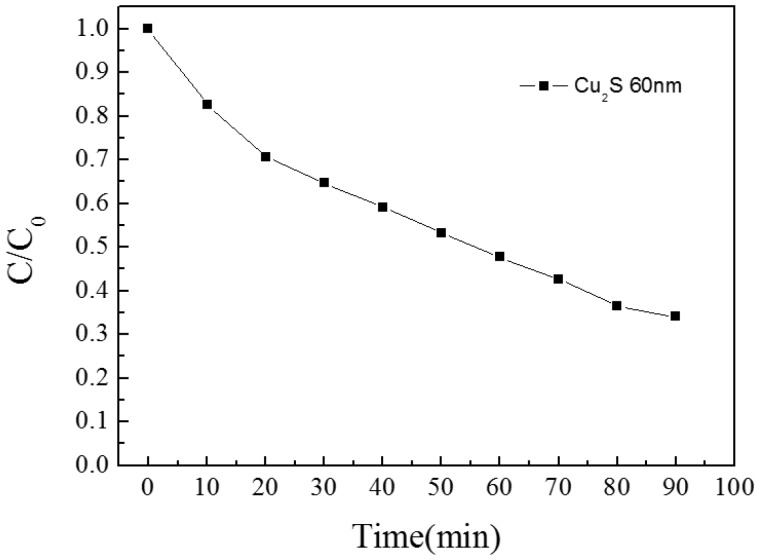
The photocatalytic activity of Cu_2_S/glass complex films for different thickness.

The results of the single Cu_2_S thin film are as follow. In Figure 5, the peak value of transmission of Cu_2_S thin film is above 60% at near 1000 nm. The sheet resistance value of single Cu_2_S thin film is 200 Ω/□ and this leads to a good conductivity of film. In Figure 6, the FOM value is first increased and then decreased, the peak value appears above 0.00005 at near 1000 nm. The experiment on photocatalytic effect of single Cu_2_S thin film was performed, and its photocatalytic efficiency value can reach to 65% (Figure 7).

**Figure 8:**
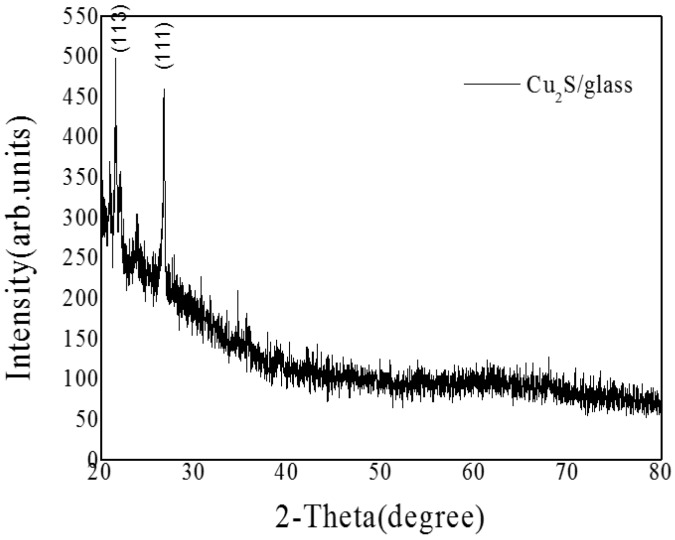
The XRD patterns of Cu_2_S/glass films.

The crystalline structures of the Cu_2_S/glass were estimated by XRD measurements, and the patterns are shown in Figure 8, where the first diffraction peak represents Cu_2_S(113), the second diffraction peak appeared at 2θ = 26.68 corresponding to Cu_2_S(111), and the lattice constant of Cu_2_S is 0.3327 nm.

The lattice mismatch (δ) between ZnO and Cu_2_S films can be calculated by the following formula:

(1) $$\delta =2\frac{\left|a_{{\mathrm{Cu}}_{2}S}-a_{\mathrm{ZnO}}\right|}{a_{{\mathrm{Cu}}_{2}S}+a_{\mathrm{ZnO}}}=2\frac{\left|0.3327-0.2602\right|}{0.3327+0.2602}=0.2475$$

The interface of ZnO and Cu_2_S films is semicoherent interface.

### 3.2. Cu_2_S/ZnO/Glass

**Figure 9:**
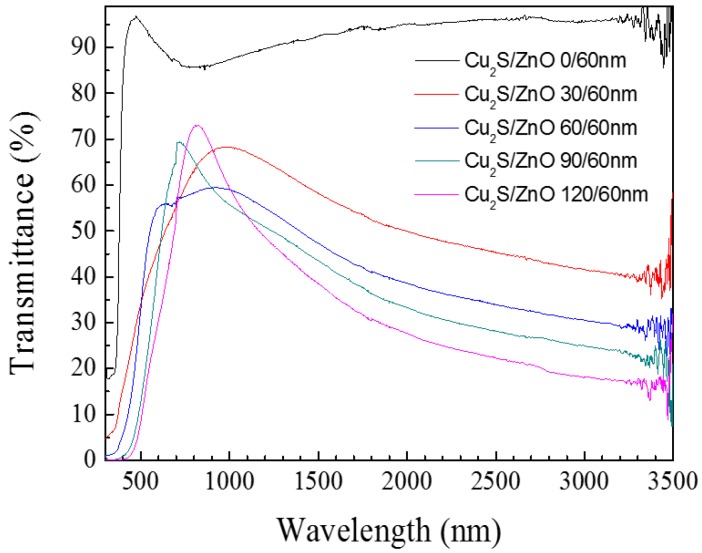
The transmittance diagram of Cu_2_S/ZnO/glass complex films for different thickness.

A series of light transmittance tests on Cu_2_S/ZnO complex films has been measured and compared with the single ZnO film. Figure 9 shows the transmittance of the Cu_2_S/ZnO complex films when the thickness of Cu_2_S increases from 0 to 120 nm. It is obvious that as the Cu_2_S thickness gradually increases, the complex film’s transmittance decreases in the infrared region, from 96% to 15% at the wavelength of 3000 nm. Simultaneously, it can be noticed that the peaks of maximum transmittance become sharper when the Cu_2_S layers become thicker. The maximum intensity of the transmission curve appears in the visible light region or in the infrared, but close to the visible region of the spectrum. The increase of the thickness of the Cu_2_S layer causes an increase of the intensity of maximum transmittance peak. We think that with the increase of the thickness of Cu_2_S in a certain extent, a better p-n junction is formed, and it also changes the structure of the composite film. With the increase of the thickness of Cu_2_S, we can see that the FOM value becomes better in the visible and near infrared regions. Additionally, for the four different thickness of the film, the transmittance maximums appear different locations.

**Figure 10:**
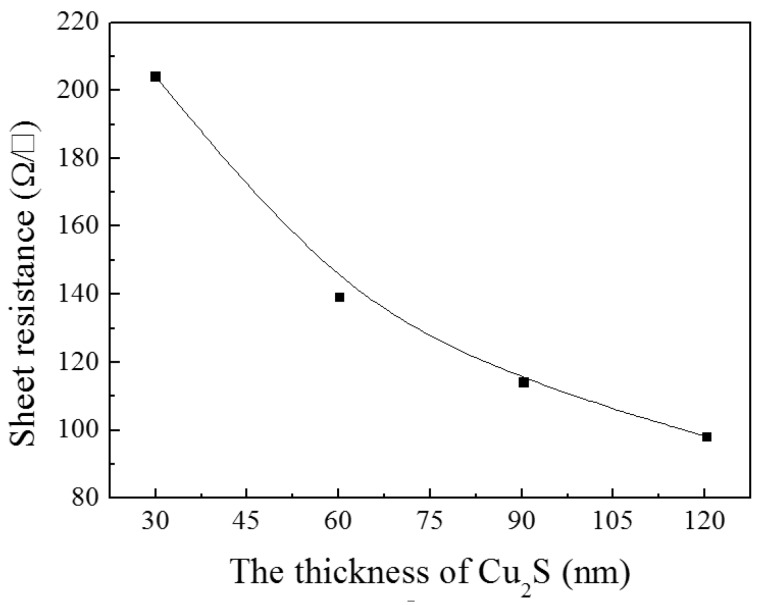
The sheet resistance of Cu_2_S/ZnO/glass complex films for different thickness.

In the resistance test, some phenomena are shown in Figure 10; it indicates that with the increase of Cu_2_S, the sheet resistance decreases. When larger amount of Cu_2_S was deposited on the film, the resistance of film declined greatly. All these can be mainly contributed to the good conductivity of Cu_2_S. With the increase of the thickness of Cu_2_S in a certain extent, a better p-n junction is formed; it is more conducive to the separation of carriers, resulting in a greater number of hole–electron pairs.

**Figure 11:**
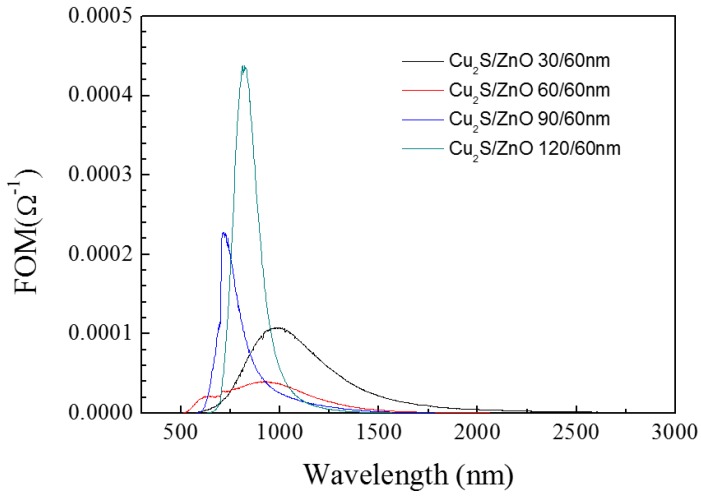
The figure of merit of Cu_2_S/ZnO/glass complex films for different thickness.

Figure 11 shows that the thickness of Cu_2_S has a great impact on FOM in visible light region. The complex film Cu_2_S (90 nm)/ZnO (60 nm) has a better FOM value. For the infrared region, the complex film Cu_2_S (120 nm)/ZnO (60 nm) has a better FOM value.

**Figure 12:**
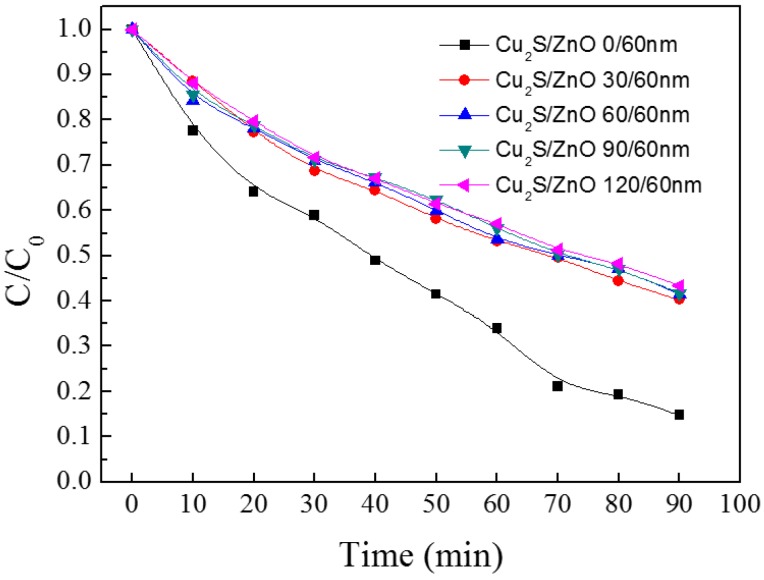
The photocatalytic activity of Cu_2_S/ZnO/glass complex films for different thickness.

The photocatalytic activities of the different Cu_2_S/ZnO films were evaluated by comparing the degradation of methyl orange under UV irradiation. In Figure 12, it is noticeable that the photocatalytic activity of the ZnO monolayer film is better than that of other complex films. The best photocatalytic efficiency of ZnO monolayer film reaches 86%. In contrast, the photocatalytic efficiency of other films just arrives at about 60%. This phenomenon can contribute to the absorbance of Cu_2_S to UV light, and the light absorbed cannot be utilized for photocatalysis instead of the vibration of phonons.

The above results show that, although the Cu_2_S film is conducive to conduction, it lowers the film catalysis efficiency and transmittance. For Cu_2_S (120 nm)/ZnO (60 nm), it has a good FOM value at the wavelength of 810 nm.

**Figure 13:**
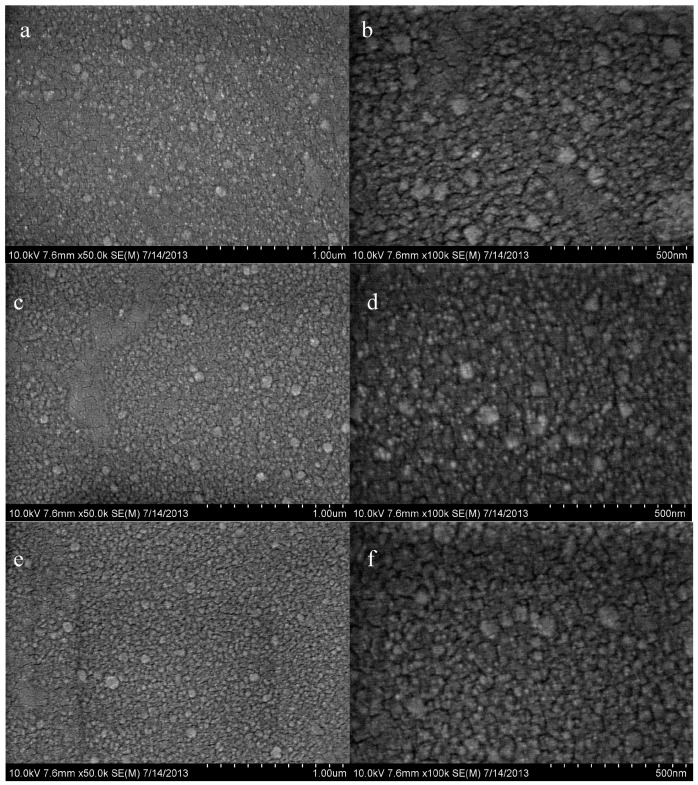
SEM micrographs of Cu_2_S/ZnO (60 nm)/glass complex films deposited with various Cu_2_S deposition thickness: (**a**) (15 nm, 7.6 mm × 50k); (**b**) (15 nm, 7.6 mm × 100k); (**c**) (30 nm, 7.6 mm × 50k); (**d**) (30 nm, 7.6 mm × 100k); (**e**) (60 nm, 7.6 mm × 50k); and (**f**) (60 nm, 7.6 mm × 100k). (50k represents the magnification, 1k = 1000).

To observe the growth behavior of Cu_2_S films, they were deposited on ZnO (60 nm)/glass with various thicknesses. Figure 13 shows the SEM micrographs of Cu_2_S/ZnO (60 nm) films with various thicknesses (15 nm, 30 nm and 60 nm) and magnification.

As can be seen in Figure 13a,b, the Cu_2_S layer deposited for 15 nm is in the form of disconnected islands. Cu_2_S islands acting as discontinuous scattering sites lead to low carrier mobility. With increasing deposition time, the grain size of Cu_2_S is increased, with thicknesses reaching 30 nm and 60 nm, the uniformity is improved, as shown in Figure 13c–f.

**Figure 14:**
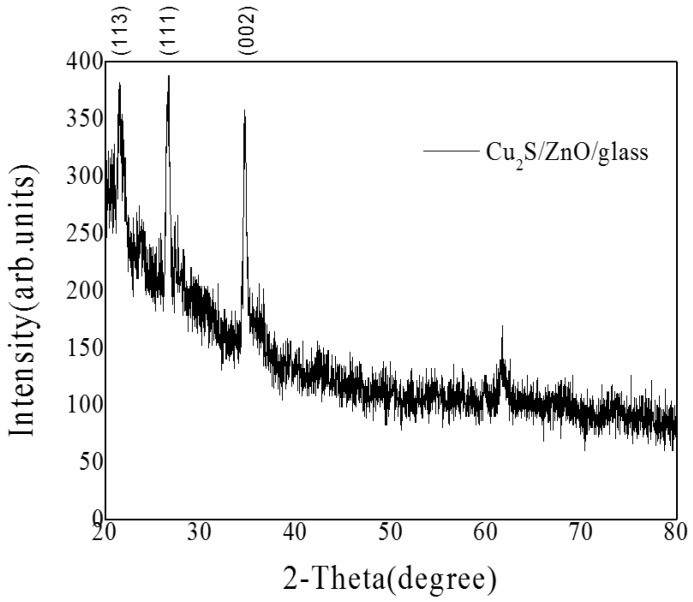
The XRD patterns of Cu_2_S/ZnO/glass films.

The crystalline structures of the Cu_2_S/ZnO/glass were estimated by XRD measurements, and the patterns are shown in Figure 14. The detected diffraction peak locates in 2θ = 26.68°, corresponding to Cu_2_S(111) and the diffraction peak locates in 2θ = 34.42°, corresponding to ZnO(002).

### 3.3. ZnO/Cu_2_S/Glass

**Figure 15:**
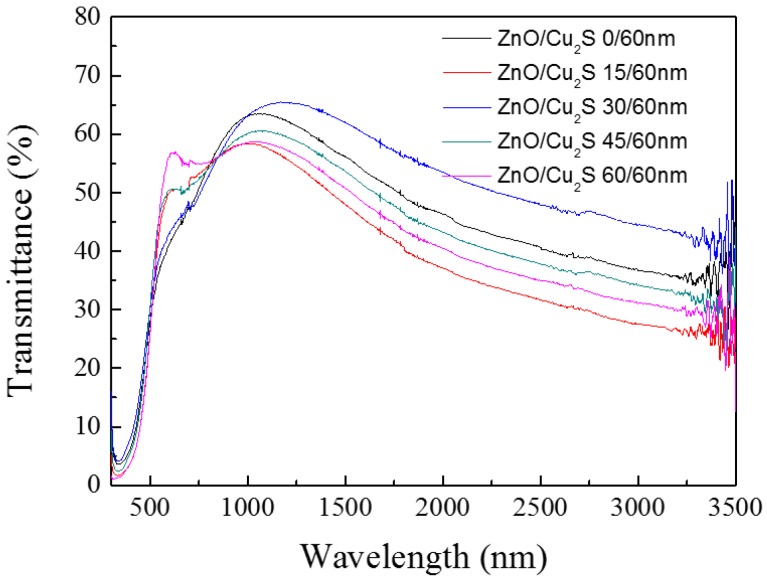
The transmittance diagram of ZnO/Cu_2_S/glass complex films for different thickness.

**Figure 16:**
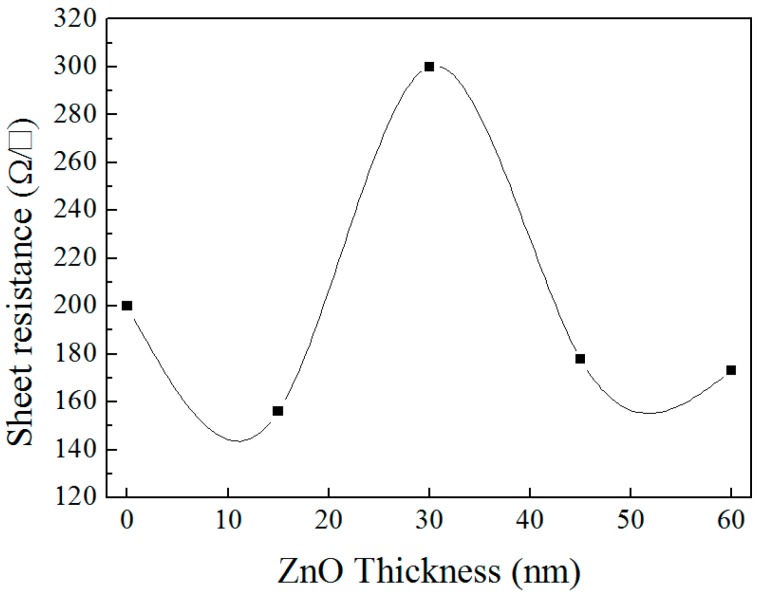
The sheet resistance of ZnO/Cu_2_S/glass complex films for different thickness.

In this section, we studied the influence of surface ZnO layer on the photoelectric performance and photocatalysis activity of ZnO/Cu_2_S complex films. ZnO layers with different thicknesses were deposited on the Cu_2_S layer of 60 nm. The transmittance spectra of ZnO/Cu_2_S complex films for different thickness are shown in Figure 15. It is obvious that with the increase of ZnO, in the visible region, the transmittance rate first increases at the thickness from 0 nm to 15 nm, then decreases at the thickness from 15 nm to 30 nm and increases again at the thickness from 30 nm to 60 nm, and in the infrared region, the tendency of the transmittance rate is consistent with the change of sheet resistance in Figure 16. We can see when the deposition thickness is 15 nm, the sheet resistance got smaller. This can be explained by the energy model and carrier transfer, due to the energy gap of ZnO is 3.37 eV, and the energy gap of Cu_2_S is 1.2 eV. When the two semiconductors contact, their Femi levels become the same. Moreover, because the ZnO is a kind of n-type semiconductor and Cu_2_S is a kind of p-type semiconductor, the p-n junction exists in the contact interface, which favors the separation of the carriers. However, as the ZnO layer becomes thicker, the carriers cannot diffuse to the surface, which leads to the result of the resistance getting bigger. However, in the observed phenomenon, a special phenomenon was observed: about the ZnO (30 nm)/Cu_2_S (60 nm) composite film, both the transmittance of the infrared spectral region and the resistance of the sheet resistance are higher than other composite films with different thickness of the ZnO.

This phenomenon is very similar to that of Cu_2_S (30 nm)/ZnO (60 nm) composite films. This is attributed to the poor combination of ZnO (30 nm)/Cu_2_S (60 nm), and is detrimental to the migration of the carriers to some extent.

The transmittance of film in the visible region mainly depends on the effect of ZnO, but in the infrared region, transmittance rate from high to low when the thickness of ZnO varies based on the order of 30nm-0nm-45nm-60nm-10nm. Thus, it can be concluded that the transmission and resistance of the complex film are connected with the thickness ratio of ZnO and Cu_2_S. When the thickness of ZnO and Cu_2_S are 30 nm and 60 nm, respectively, at the wavelength of 1168 nm, both transmission and resistance have maximum values.

**Figure 17:**
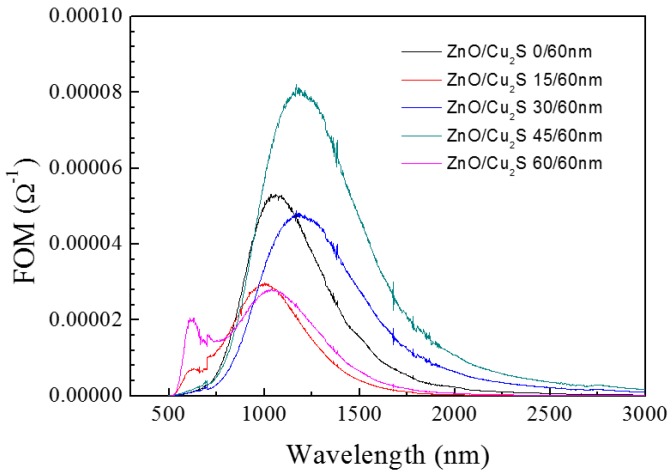
The figure of merit of ZnO/Cu_2_S/glass complex films for different thickness.

**Figure 18:**
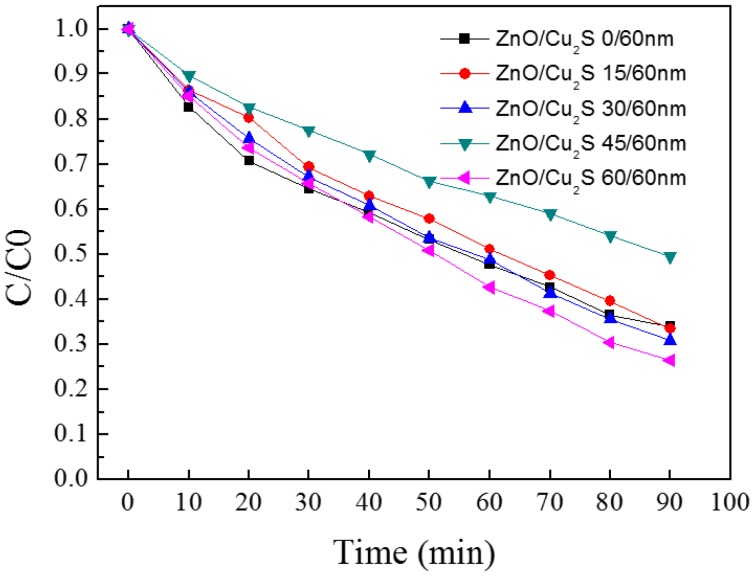
The photocatalytic activity of ZnO/Cu_2_S/glass complex films for different thickness.

Figure 17 shows the figure of merit of ZnO/Cu_2_S complex films for different thickness. It is obvious that in the visible light region, when the ZnO is 60 nm thick, the film has a better FOM value than that of others and when the ZnO is 45 nm thick, in the infrared region, the value gets bigger. In the meantime, we can see between the visible light and infrared regions, the curves roughly present an opposite trend. Moreover, it shows the tendency that films with good FOM values have better photocatalytic activity in the visible light region. The change of photocatalytic activity is displayed in Figure 18. The photocatalytic efficiency varies from 50% to 75%. When the deposition thickness is 45 nm, photocatalytic efficiency is worst among the five samples, and when the thickness is 60 nm, the best photocatalytic efficiency is approximate to 75%. Here, as for the variance between photocatalytic activities, we can see that when the thickness of top layer ZnO is 60 nm, the photocatalytic activity of ZnO/Cu_2_S complex films is the best. The thickness of ZnO has an effect on photocatalytic activity of ZnO/Cu_2_S complex films, and its effect appears only when the thickness is above a threshold.

**Figure 19:**
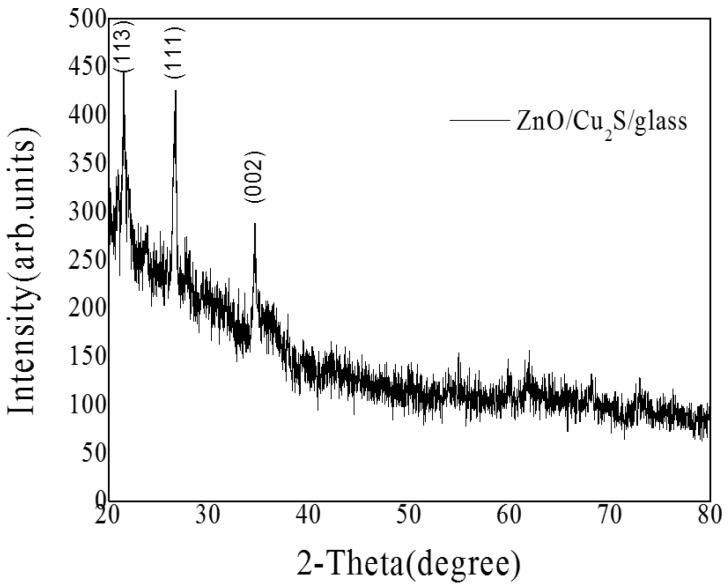
The XRD patterns of ZnO/Cu_2_S/glass films.

The crystalline structures of the ZnO/Cu_2_S/glass were estimated by XRD measurements, and the patterns are shown in Figure 19. The detected diffraction peak locates in 2θ = 26.68°, corresponding to Cu_2_S(111) and the diffraction peak locates in 2θ = 34.42°, corresponding to ZnO(002).

### 3.4. ZnO/Cu_2_S/ZnO/Glass

In order to guide the experiments in theory, numerical simulations of the optical properties of multilayer films were carried out. The characteristic matrix ***M*** of the stratified medium given by Born [22] was used in this computer program. The characteristic matrix of the whole ZnO/Cu_2_S/ZnO/glass multilayer film can be expressed as:

(2) $$M=M_{\mathrm{ZnO}}\left(n_{1},k_{1}\right)M_{{\mathrm{Cu}}_{2}S}\left(n_{2},k_{2}\right)M_{\mathrm{ZnO}}\left(n_{3},k_{3}\right)$$

where ***n_i_*** and ***k_i_*** are the refractive index and extinction coefficient, respectively of corresponding medium layer.

For each combination of Cu_2_S and ZnO layer thicknesses in the ZnO/Cu_2_S/ZnO/glass films, the values of the transmittance at normal incidence are calculated using the equation for T [22].

In the simulation, the optical constants *n* and *k* for the ZnO and Cu_2_S are very important. The constants ***n*** and ***k*** for the ZnO can be used from the AvaSoft software (Avaspec-2048FT-2-SPU, Avantes, Apeldoom, Holland), but under present conditions, we do not have the comprehensive description of the optical constants of the Cu_2_S. In this situation, we use the modified optical constants of the ZnS to replace the constants of the Cu_2_S, and the modified constants are used for the numerical simulation of the optical properties of the ZnO/Cu_2_S/ZnO multilayer films. In order to get the simulated transmission spectra of three-layer structure more comprehensively, the transmission spectra of bilayer structure were simulated in advance.

**Figure 20:**
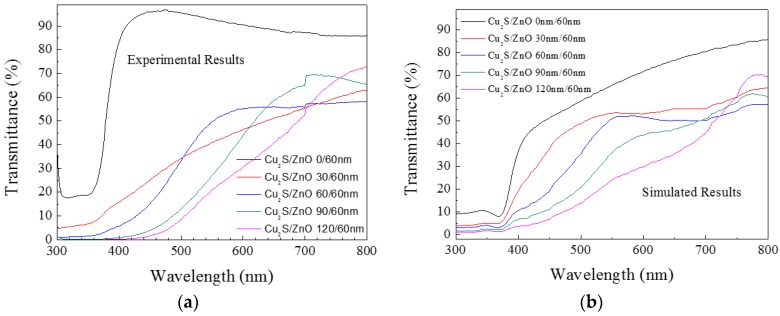
(**a**) is experimental results of Cu_2_S/ZnO/glass film and (**b**) is simulated results of Cu_2_S/ZnO/glass film.

**Figure 21:**
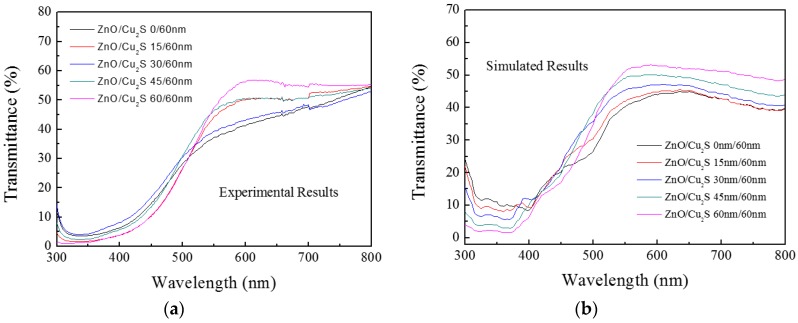
(**a**) is experimental results of ZnO/Cu_2_S/glass film and (**b**) is simulated results of ZnO/Cu_2_S/glass film.

A number of theoretical simulations have been carried out on ZnO/Cu_2_S/glass and Cu_2_S/ZnO/glass bilayers in this investigation. It is found that the variations in simulated optical transmittance are generally consistent with the experimental results. The results of transmittance are displayed in Figure 20 and Figure 21.

After the research on two kinds of structure films, Cu_2_S/ZnO/glass and ZnO/Cu_2_S/glass, we further investigate the influence of ZnO/Cu_2_S/ZnO/glass interference on transmission, conductivity and photocatalysis to investigate the function of top ZnO by theoretical simulation and experimental results.

**Figure 22:**
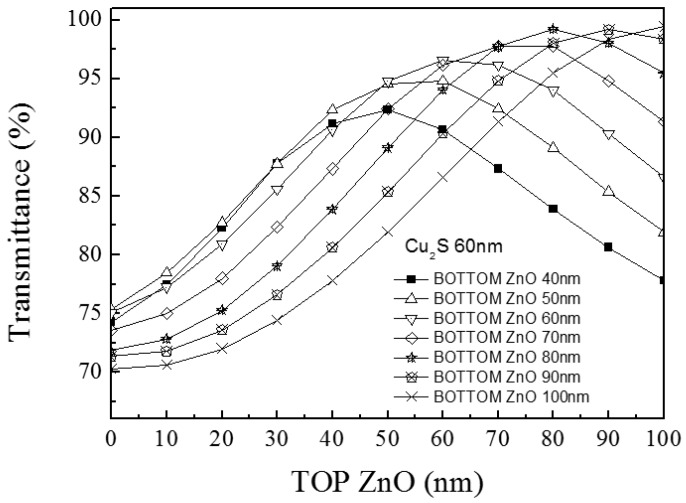
Comparison between simulation and experimental transmission spectrum of ZnO (30 nm)/Cu_2_S (60 nm)/ZnO (60 nm)/glass multilayer and ZnO (60 nm)/Cu_2_S (60 nm)/ZnO (60 nm)/glass multilayer.

Dependence of the simulated maximum transmittance of the multilayer on the thickness of top ZnO layer in the condition of fixed middle Cu_2_S and bottom ZnO layer thicknesses for each curve is shown in Figure 22. At the fixed Cu_2_S thickness (60 nm), the seven curves represent seven different thicknesses of the bottom ZnO layers (40–100 nm, spacing for 10 nm) in this graph. The wavelength which corresponds to maximum transmittance is not a fixed value but each wavelength corresponds to the maximum transmittance value. It is observed that when the top ZnO thickness is or nearly equal to bottom ZnO thickness, the transmittance reaches a maximum value.

**Figure 23:**
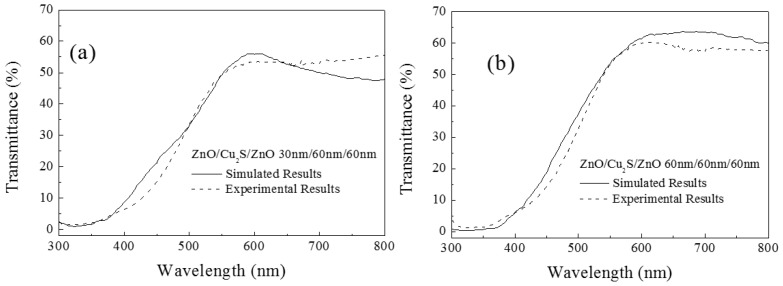
The simulation maximum transmittance of the multilayer on the thickness of top ZnO layer (the thicknesses of middle Cu_2_S is 60 nm and bottom ZnO layer thicknesses is 60 nm for each curve).

A number of theoretical simulations have been carried out in this investigation: it is found that the variations in simulated optical transmittance are generally consistent with the experimental results. Some simulated transmission spectra agreed with the experimental spectra. For example, Figure 23 gives a good result about the comparison between simulation and experimental transmission spectrum of ZnO (30 nm)/Cu_2_S (60 nm)/ZnO (60 nm) multilayer (Figure 23a) and ZnO (60 nm)/Cu_2_S (60 nm)/ZnO (60 nm) multilayer (Figure 23b).

**Figure 24:**
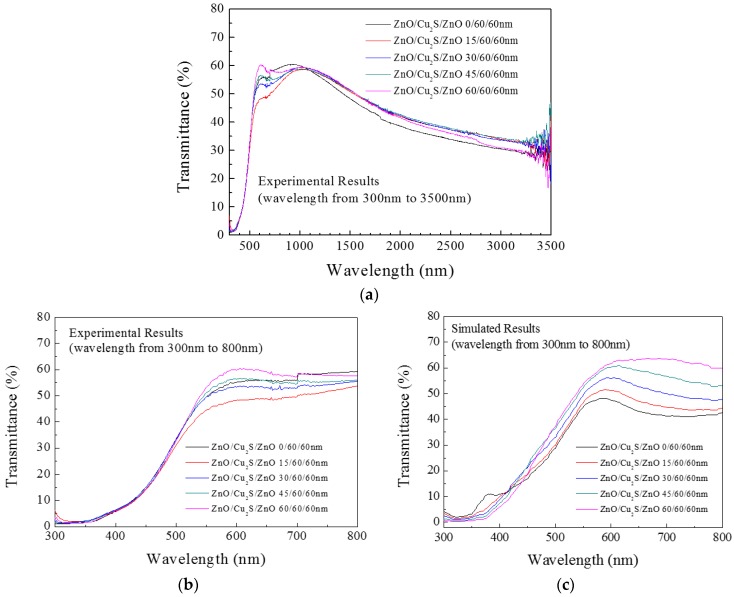
The (**a**,**b**) experimental and (**c**) simulation transmittance results of ZnO/Cu_2_S/ZnO/glass complex films for different thickness.

In the transmission of complex films, Figure 24 shows the transmittance of the ZnO/Cu_2_S (60 nm)/ZnO (60 nm) complex films as the thickness of top layer ZnO increased from 0 to 60 nm. As the ZnO thickness generally increased, the complex film’s transmittance decreased in the infrared region, from 48% to 27% at the wavelength of 3250 nm. In the visible light region, when the ZnO film gets thicker, the transmittance gets bigger. Additionally, for the four different film thicknesses, in the visible light region, the partial maximum value of each transmittance appears in the same location, near the wavelength of 600 nm, and the partial maximum value of transmittance corresponds to the maximum thickness. The phenomenon can be attributed to the film interference effect [3,23]. Compared with dual-structure films, the peak values of transmittance of three layers films appeared in visible region; in infrared region, the property of transmission is affected a little by top ZnO, the transmittance curves comparatively resemble with others.

**Figure 25:**
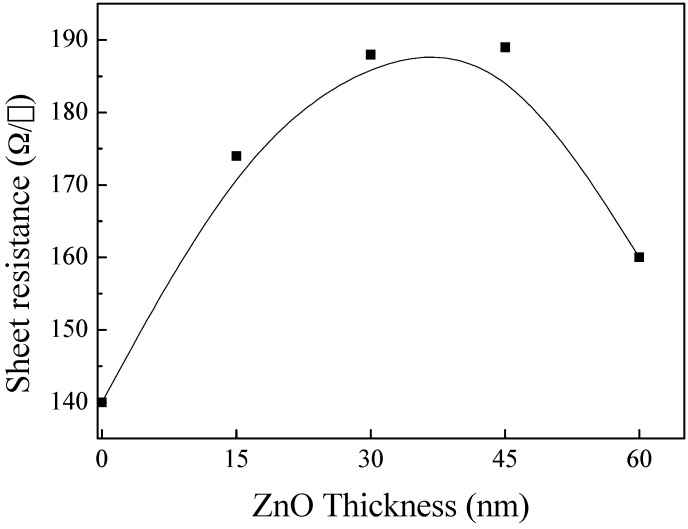
The sheet resistance of ZnO/Cu_2_S/ZnO/glass complex films for different thickness.

In the research on resistance property, Figure 25 indicates that with the increase of ZnO, the variation of the sheet resistance resemble a shape of parabola. The trend of the curve presents firstly increase and then decrease, and the maximum sheet resistance appears at 45 nm and a marked decline appears at 60 nm, which is in accordance with the infrared region in Figure 23. Three-layer structures can realize the goal to limit sheet resistance under threshold value (for example, 190 Ω/□).

**Figure 26:**
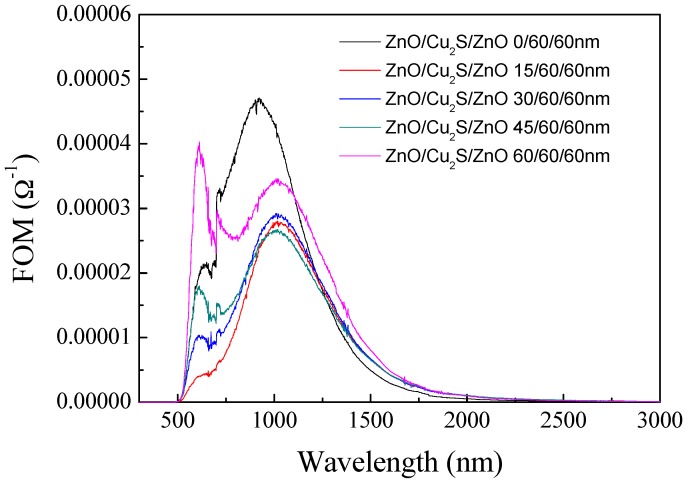
The figure of merit of ZnO/Cu_2_S/ZnO/glass complex films for different thickness.

The figure of merit of ZnO/Cu_2_S/ZnO/glass complex films for different thickness is shown in Figure 26. It is obvious that in the visible light and infrared regions, as the ZnO layer thickness increases, the film has much better FOM value.

**Figure 27:**
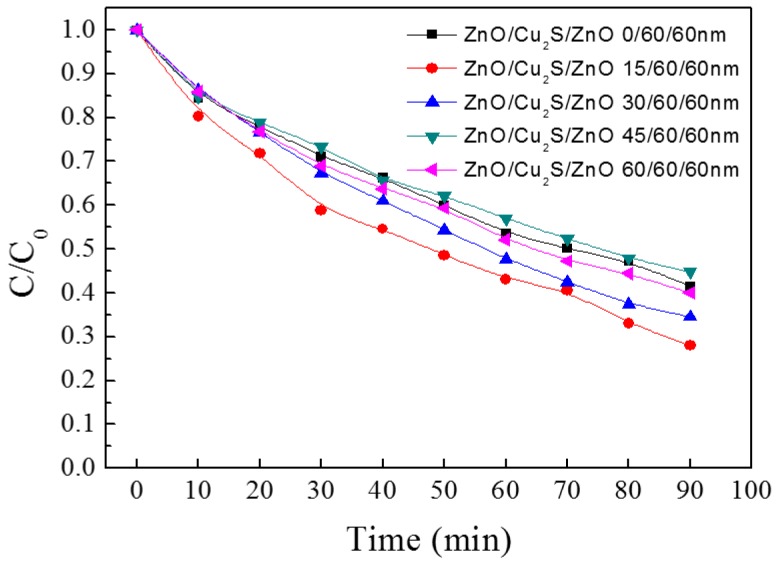
The photocatalytic activity of ZnO/Cu_2_S/ZnO/glass complex films for different.

The change of photocatalytic activity is exhibited in Figure 27. The degradation rates vary from 55% to 72%. When the thickness of top layer ZnO is 45 nm, the degradation rate is worst among the five samples, and when the thickness of top layer ZnO is 15 nm, the best degradation rate is achieved, approximately 72%. Here, as for the variance between photocatalytic activities, it is noticeable that the thickness of top layer ZnO is 15 nm, the photocatalytic activity of ZnO/Cu_2_S/ZnO complex films is best. It can be found that there is the certain relationship between the FOM and the degradation rate: the smaller the FOM value is, the better the photocatalytic activity is.

**Figure 28:**
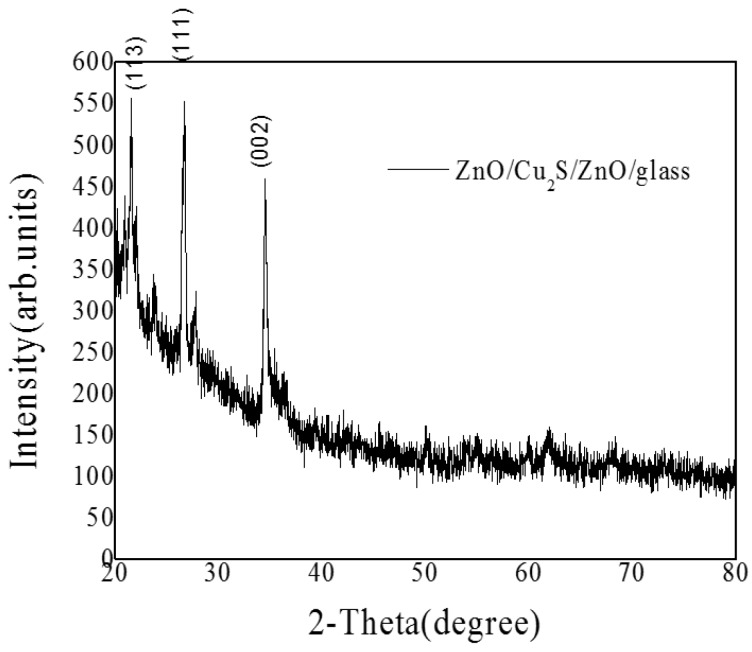
The XRD patterns of ZnO/Cu_2_S/ZnO/glass films.

The crystalline structures of the ZnO/Cu_2_S/ZnO/glass were estimated by XRD measurements, the patterns are shown in Figure 28. The detected diffraction peak locates in 2θ = 26.68°, corresponding to Cu_2_S(111) and the diffraction peak locates in 2θ = 34.42°, corresponding to ZnO(002).

## 4. Conclusions

Because of the interaction of ZnO and Cu_2_S, the complex films show us a series of good results. From the three groups of experiments results, some characteristics can be concluded. In comparison with pure ZnO film, although the optical transmittance and the photocatalytic properties of the Cu_2_S/ZnO/glass complex film are slightly lowered, the electrical conductivity is remarkably increased. Similarly, compared with pure ZnO film, the transmittance and photocatalytic activity of the ZnO/Cu_2_S/glass complex film performs slightly lower, but the conductivity is clearly improved.

Compared with single Cu_2_S film, the transmission property and photocatalytic performance of ZnO/Cu_2_S/glass film have remarkably improved. The optimal photocatalytic efficiency of Cu_2_S film is about 65%; however, the optimal photocatalytic efficiency of ZnO/Cu_2_S/glass film is approximately 75%, and the photocatalytic efficiency of ZnO/Cu_2_S/ZnO/glass film is more than 70%. In addition, ZnO/Cu_2_S/ZnO/glass complex film has a much higher electrical conductivity compared to single ZnO film. The research also proves that different complex film component ratios have obvious effects on the properties of film. When the complex film component ratio is optimized, excellent performance of complex films can be achieved.
